# A multivalent subunit vaccine augments pre-existing immunity to protect against T3SS-positive *Pseudomonas aeruginosa* but reveals immunological interference against ExlA-positive strains

**DOI:** 10.1128/iai.00175-26

**Published:** 2026-08-04

**Authors:** Debaki R. Howlader, Sayan Das, Satabdi Biswas, Zackary K. Dietz, Robert K. Ernst, William D. Picking, Wendy L. Picking

**Affiliations:** 1 Department of Pathobiology & Integrative Biomedical Sciences and the Bond Life Sciences Center, University of Missouri 14716, Columbia, MO, USA; 2 Department of Microbial Pathogenesis, University of Maryland 1068 , College Park, MD, USA; Tsinghua University, Beijing, China

**Keywords:** *Pseudomonas*, pre-exposure, vaccine, T3SS, subunit vaccine, L-PaF/E/ME/BECC

## Abstract

*Pseudomonas aeruginosa* (Pa) is a ubiquitous, opportunistic nosocomial pathogen that poses a significant threat due to its innate and acquired multidrug resistance. Novel vaccine strategies are urgently needed for vulnerable populations, many of which harbor pre-existing immunity from prior encounters with Pa. Here, we evaluated a multivalent subunit vaccine combining type III secretion system (T3SS) antigens and exolysin A (ExlA) in a nanoemulsion formulation using a clinically relevant murine pulmonary pre-exposure model. This approach allowed us to determine whether vaccination could overcome the limitations of the host’s initial, ineffective immune response. Vaccination fundamentally transforms suboptimal baseline memory into a potent, multi-faceted Th1/Th17-polarized response. This globally transformed signature was characterized by significantly enhanced antigen-specific IFN-*γ* and IL-17A production, both locally and systematically in the lung, with exceptionally large biological effect sizes (Cohen’s *d* values reaching 21.35). By utilizing log10 transformation to accurately reflect pathogen growth kinetics, we demonstrated that this vaccine-augmented immunity conferred statistically significant protection following heterologous challenge. In the twice-exposed cohort, vaccination promoted superior bacterial clearance of the T3SS-positive strain, though clearance of the ExlA-positive strain was not enhanced, potentially due to immunological interference from pre-existing T3SS memory. In the thrice-exposed cohort, a functional protective threshold was observed, where high levels of natural immunity matched the vaccine-induced clearance levels. Our findings established that our vaccine formulation can effectively boost and redirect pre-existing immunity, offering a promising approach to overcome the limitations of natural exposure and protect at-risk individuals from diverse Pa infections.

## INTRODUCTION

The opportunistic pathogen *Pseudomonas aeruginosa* (Pa) represents a formidable global health challenge, primarily owing to severe nosocomial infections in vulnerable populations. Immunocompromised individuals, the elderly, and patients with underlying conditions, including cystic fibrosis (CF) or those requiring mechanical ventilation, are at significant risk of Pa infection (1, 2). The US Centers for Disease Control and Prevention (CDC) has classified multi-drug-resistant (MDR) Pa as a serious threat, responsible for an estimated 32,600 hospitalizations and 2,700 deaths in the United States annually, with associated healthcare costs approaching $757 million (3). Beyond mortality, this pathogen imposes a substantial burden on quality of life, evidenced by a disability-adjusted life year (DALY) impact of 2.81 × 10⁻⁹ per event and its role in causing chronic, debilitating lung disease in over 70% of individuals with CF (4, 5).

The increasing prevalence of MDR and extremely drug-resistant (XDR) Pa strains (6) makes the development of a broadly protective vaccine a critical unmet medical requirement (7). Historical vaccine strategies targeting serotype-specific lipopolysaccharide (LPS) or using whole-cell formulations have been hampered by the limited breadth of coverage and the risk of reactogenicity, particularly in immunocompromised patients (8, 9). These limitations have directed research toward subunit vaccines that target highly conserved virulence factors. Among these are components of the type III secretion system (T3SS), a molecular syringe required for the pathogenesis of the majority of Pa strains, with the needle tip protein PcrV and translocator PopB being prime targets for inducing serotype-independent immunity (10–12). However, a truly comprehensive vaccine must also address the emergence of clinically relevant T3SS-negative strains that utilize an alternative virulence factor, exolysin A (ExlA), indicating the need for a multivalent vaccine approach to ensure broad protection (13).

A prophylactic vaccine is a major step forward in combating Pa infections, as the immunity derived from natural exposure is often suboptimal and carries a significant risk of severe clinical complications. While natural infections can establish immunological memory, this benefit is offset by the risk of significant morbidity, clinical sequelae, and a high DALY burden, especially in the elderly (14). Moreover, severe bacterial infections can trigger an overwhelming inflammatory response that paradoxically further dampens the immune system (15). This includes inducing a state of “endotoxin tolerance” in innate cells, rendering them unresponsive to subsequent microbial threats, and driving adaptive T cells into a dysfunctional state of exhaustion characterized by inhibitory receptors, such as PD-1 (16). This cellular exhaustion, compounded by the expansion of regulatory cells and suppressive cytokines, culminates in a systemic immunodeficiency that impairs recovery and increases susceptibility to secondary infections. Despite the ubiquity of *Pseudomonas aeruginosa* and the high prevalence of natural exposure, these prior encounters typically fail to elicit protective immunity, leaving vulnerable populations at risk. This highlights the urgent need for a vaccine that can overcome the limitations of natural exposure.

Here, we assessed the immunogenicity and protective efficacy of a multivalent subunit vaccine in a clinically relevant murine model designed to simulate prior, escalating bacterial encounters. We demonstrated that vaccination of these pre-exposed hosts drives the maturation of suboptimal baseline memory into a potent and multi-faceted Th1/Th17-polarized immune response. This globally transformed signature is characterized by robust, antigen-specific cytokine production with high biological effect sizes that effectively override the ineffective pre-existing immune state. We show that this vaccine-augmented immunity confers statistically significant protection, as evidenced by enhanced bacterial clearance from the lungs following challenge with two phenotypically distinct isolates: the mucoid, T3SS-positive strain mPa08-31 and the non-mucoid, ExlA-positive strain CEC124. Furthermore, we established that the magnitude of this protective efficacy is contingent upon the host’s prior exposure history and is associated with the pre-challenge immunological state, highlighting a potential correlation between protection against T3SS-mediated virulence. Collectively, this work provides a comprehensive pre-clinical validation of a vaccine strategy capable of effectively boosting and redirecting pre-existing immunity to confer protection against diverse and heterologous *Pa* strains.

## MATERIALS AND METHODS

### Animals

Female BALB/c mice (6–8 weeks old) were procured from Charles River Laboratories (Wilmington, MA, USA). BECC438b is a biologically derived lipid A analog that is a potent TLR-4 agonist and adjuvant (17). In all cases, mice were anesthetized using 5% isoflurane in a sealed induction chamber in the presence of oxygen at a displacement rate of 3 L/min.

### Bacterial strains and inoculum preparation

Infection models utilized two distinct clinical isolates of Pa: the mucoid, T3SS-positive strain mPa08-31, and T3SS-negative strain CEC124. For experimental use, challenge doses were prepared by initially cultivating the strains from cryopreserved stocks on *Pseudomonas* Isolation Agar (PIA). Subsequently, individual colonies were propagated in low-salt Luria-Bertani (LB) broth overnight at 37°C with agitation. On the day of experiments, these stationary-phase cultures were diluted into fresh medium and cultivated to a mid-logarithmic growth phase (OD_600_ ≈ 0.3) before being harvested and resuspended in PBS for pre-exposure and challenge studies.

### Generation of recombinant antigens and vaccine assembly

Recombinant antigens were generated and subsequently assembled into a nanoemulsion-based vaccine formulation as outlined below.

#### L-PaF antigen synthesis

Expression of the L-PaF fusion protein (LTA1-PcrV-PopB) was in *E. coli* Tuner cells using a fed-batch fermentation system. LTA1 refers to the A1 subunit of the heat-labile enterotoxin from *Escherichia coli*. The purification strategy involved an initial chaperone-assisted capture of the L-PaF/His6-PcrH complex via Immobilized Metal Affinity Chromatography (IMAC), followed by a dissociation step using the detergent LDAO to liberate the target antigen (10).

#### ExlA antigen synthesis

Recombinant expression of the C-terminal domain of Exolysin A (residues 1,361–1,651) was performed in *E. coli* BL21 (DE3). This specific fragment was selected as it contains the critical domain required for host cell membrane disruption but remains functionally inactive/non-hemolytic at physiological pH as a purified subunit. For nomenclature consistency with our previous work (13), this C-terminal subunit is referred to as ExlA throughout this study. The His-tagged protein was purified using IMAC, after which a stringent endotoxin-depletion protocol involving iterative Triton X-114 phase separation was employed to reduce LPS levels to <5 EU/mg (13, 18).

#### Vaccine platform formulation

The vaccine delivery vehicle was a squalene-based (8% wt/vol) oil-in-water nanoemulsion stabilized with polysorbate 80 (2% wt/vol) (19, 20). This nanoemulsion Medimmune emulsion (ME) was fabricated via high-pressure microfluidization to achieve a uniform particle size. The final immunizing agent was generated by integrating the L-PaF and ExlA antigens with the ME vehicle and the TLR4 agonist, BECC438b. One microgram of L-PaF and ExlA, along with 0.5 mg of BECC438b in ME, was used per mouse for all mouse studies.

### *In vivo* pre-exposure and immunization regimen

**Fig 1:**
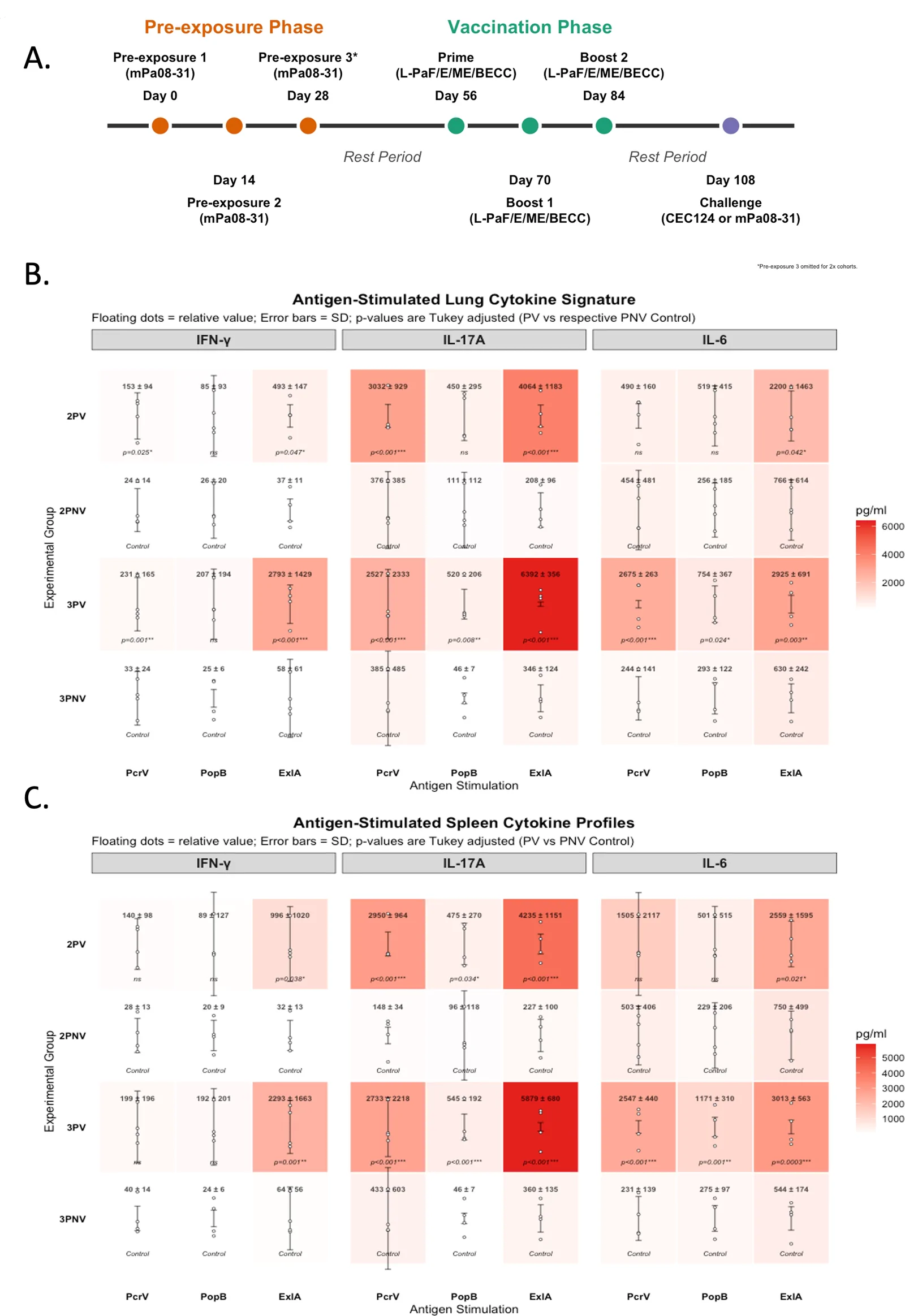
Experimental schema and vaccine-induced antigen-specific cytokine recall response in both pulmonary and systemic compartments. (**A**) Schematic representation of the study design and immunization timeline. Mice received two or three escalating pulmonary pre-exposures to *Pa* mPa08-31 on Days 0, 14, and 28. Following a 28-day rest period, mice were immunized intranasally with the multivalent L-PaF/E/ME/BECC vaccine or PBS on Days 56, 70, and 84. Final challenge and immunological assessments were conducted on Day 108. Single-cell suspensions from the (**B**) lung and (**C**) spleen of pre-exposed mice were harvested pre-challenge and stimulated *in vitro* for 48 h with recombinant antigens (PcrV, PopB, or ExlA). Concentrations (pg/mL) of IFN-*γ*, IL-17A, and IL-6 in culture supernatants were quantified by multiplex immunoassay. Data are visualized as an integrated heatmap where background color intensity corresponds to the group means. Each tile provides a comprehensive data profile: the upper text indicates the mean ± standard deviation (SD); central white circles represent individual biological replicates (*n* = 4) positioned vertically according to relative concentration with error bars representing the SD; and the lower text displays the adjusted *P*-value. Statistical significance was determined using a two-way ANOVA followed by Tukey’s post hoc test. The *P*-values embedded within the vaccinated (PV) blocks represent the statistical comparison against their respective non-vaccinated (PNV) control groups, which serve as the baseline for evaluating vaccine-induced recall. *P < 0.05, **P < 0.01, ***P < 0.001.

To accurately mimic the natural phenomenon in which the general population is far more likely to encounter T3SS-positive strains, which represent most of the described clinical Pa isolates, we utilized a T3SS-expressing strain in our pulmonary pre-exposure model. We pre-exposed female BALB/c mice intranasally (IN) to mPa08-31. Two distinct cohorts were established: one receiving two pre-exposures to the T3SS-positive strain mPa08-31 (1 × 10⁵ and 1 × 10⁶ CFU/mouse) and another receiving three escalating pre-exposures to the same Pa strain (1 × 10⁵, 1 × 10⁶, and 1 × 10⁷ CFU/mouse) at 14-day intervals. Following this pre-exposure phase and rest period, the vaccinated cohorts (2PV and 3PV) received a prime-boost-boost IN immunization (with 1 μg of each protein) series with the multivalent vaccine at 14-day intervals, beginning on day 56 post-initial exposure. Control cohorts received PBS, but were similarly pre-exposed either twice (2PNV) or three times (3PNV). A schematic is shown in Fig. 1A.

### Assessment of protective efficacy via bacterial challenge in mice

To evaluate the breadth of vaccine-mediated protection across diverse clinical phenotypes and distinct virulence strategies, mice were challenged with either the T3SS-positive mucoid strain mPa08-31 (4 × 10⁷ CFU/mouse) or the T3SS-negative, ExlA-producing non-mucoid strain CEC124 (1 × 10⁷ CFU/mouse). The endpoint for bacterial quantification was 24 h post-infection for CEC124 and 48 h for mPa08-31 (13). Lungs were aseptically harvested, homogenized, and serially diluted for plating on PIA to determine the bacterial burden, and the results were expressed as CFU/lung.

### Assessment of cellular and humoral immune responses

We characterized vaccine-induced cellular and humoral immune responses to elucidate the immunological mechanisms underlying the observed bacterial clearance.

#### Antigen-specific T-cell responses

The frequency of antigen-specific T cells was investigated using a dual-color IFN-γ/IL-17A ELISpot assay (10–13). Single-cell suspensions from murine lungs and spleens were stimulated with recombinant PcrV, PopB, or ExlA (5 µg/mL) for 24–48 h. The number of spot-forming cells (SFCs) per million input cells was quantified using an automated ELISpot reader.

#### Quantification of secreted cytokines

The profile of secreted effector molecules was quantified from culture supernatants derived from both pre-challenge stimulated and post-challenge unstimulated lung cells (10–13). Cytokine concentrations (IFN-γ, IL-17A, and IL-6) were determined using a U-PLEX multiplex immunoassay on a Meso Scale Discovery (MSD) platform.

#### ELISA

Briefly, 96-well plates were coated with 1 µg/mL of PcrV or PopB or LPS extracted from mPa08-31 for 3 h and subsequently blocked overnight at 4°C with 10% non-fat dry milk. Mouse serum was then added and incubated for 1 h at 37°C before washing with PBS-Tween 20. Detection was achieved by adding HRP-conjugated goat anti-mouse IgG secondary antibodies (1:1,000, Sera Care, cat # 5450-0011/474-1806), followed by TMB substrate. The reaction was stopped with phosphoric acid, and endpoint titers were reported as ELISA units per mL (EU/mL) (10–13). ExlA was not included here because all pre-exposures were conducted with mPa08-31.

### Data analysis and statistics

Graphs were prepared using R (v.4.5) and MetaboAnalyst (v.6.0). The distribution of data was first assessed for normality using the Shapiro-Wilk test. Statistical significance for multivariate cytokine, cellular, and log10-transformed bacterial clearance (CFU) datasets was determined using two-way ANOVA, followed by Tukey’s post hoc test for multiple comparisons. The suitability of parametric testing was confirmed by evaluating the homogeneity of variance across the experimental groups. For specific pairwise comparisons of bacterial burdens where indicated, an unpaired t-test on log10-transformed values was utilized. The relationships between immunological parameters and pathogen burden were evaluated using Pearson’s correlation analysis.

To supplement frequentist *P*-values and provide a more comprehensive assessment of biological significance, effect sizes were calculated for all the primary comparisons. Cohen’s *d (d*) was utilized as the standardized mean difference metric, as it allows for a direct, unit-independent comparison of the magnitude of the vaccination effect across disparate parameters, including cytokine concentrations, T-cell frequencies, and bacterial burdens. The overall variance explained by vaccination and stimulation was assessed using partial eta squared (*η_p_*^2^). Effect sizes were interpreted according to the following standard benchmarks: small (*d* ≈ 0.2), medium (*d* ≈ 0.5), large (*d* ≥ 0.8), and huge (*d* ≥ 2).

The non-vaccinated (PNV) groups served as the baseline biological controls for all statistical comparisons. Unless otherwise specified, data represent four to five biological replicates per group and are expressed as mean ± SD. Statistical significance was indicated as follows: *P < 0.05, **P < 0.01, and ***P < 0.001. Detailed statistical summaries, including adjusted *P*-values and effect size estimates, are provided within the respective figure panels and legends.

## RESULTS

### Naturally acquired humoral immunity evolves distinctly during escalating pulmonary pre-exposure

To characterize the pre-existing humoral immune response elicited by prior bacterial encounters, we quantified the serum antibody titers against the principal vaccine antigens during the pre-exposure phase. Longitudinal analysis of absolute serum IgG titers (EU/mL) showed a clear kinetic evolution across both pre-exposure cohorts (Fig. S1A). These profiles represent the baseline immunity established by the whole-cell mPa08-31 strain before initiation of the vaccination regimen. Notably, while antibody levels against the protein antigens PcrV and PopB were induced by the initial infections, they followed a gradual downward trend over the 56-day period. In contrast, anti-LPS titers exhibited a robust and progressive increase, reflecting the cumulative immune priming elicited by repeated whole-cell pre-exposures. This visualization of absolute magnitude provides a transparent baseline of the host’s “natural” immune state, confirming that the subsequently administered multivalent vaccine enters a biological environment already characterized by significant, albeit waning, anti-protein immunity and rising anti-LPS titers.

### L-PaF/E/ME/BECC enhances antigen-specific Th1/Th17 cytokine secretion in lungs and spleen of pre-exposed mice

Having characterized the baseline humoral landscape established by natural pre-exposure, we next evaluated the capacity of the multivalent vaccine to fundamentally remodel and augment antigen-specific cellular recall responses in these primed hosts. To characterize the functional capacity, breadth, and antigen specificity of the vaccine-boosted cytokine responses in both the local (lung) and systemic (spleen) compartments, we performed *in vitro* re-stimulation of isolated lung cells with PcrV, PopB, and ExlA. Vaccinated mice pre-exposed to Pa two or three times (2PV and 3PV, respectively) exhibited a markedly superior cytokine recall response compared with the non-vaccinated control groups (PNV) (Fig. 1B and C). These differences were characterized by consistently large-to-huge effect sizes (Cohen’s *d* > 0.8) across all three cytokines. Notably, the magnitude of the vaccine-induced IL-17A response in 3PV lungs following ExlA stimulation was exceptionally high, reaching an effect size of *d* = 21.35 (Fig. 1B), indicating a biologically robust Th17 signature. To further dissect the local inflammatory environment, we performed a correlation analysis of pulmonary cytokine concentrations, which revealed a tightly co-regulated network with substantial positive correlations (many *R*-values > 0.8) between IFN-*γ*, IL-17A, and IL-6 (Fig. S1B). Systemic cytokine production in the spleen followed a similar trajectory, with vaccination eliciting significant increases in IL-17A and IL-6 production (*d* values ranging from 1.14 to 7.10) (Fig. 1B). To confirm that this was a *bona fide* antigen-driven event, we assessed parallel unstimulated control cultures that showed negligible cytokine production and low effect size magnitudes (*d* primarily <0.8) across all groups (Fig. S2), establishing the high antigen specificity of the vaccine-induced recall. This shows that the vaccine-augmented immune signature is characterized by a balanced and coordinated secretion of multiple effector cytokines.

### L-PaF/E/ME/BECC expands the pool of antigen-specific T cells in the lungs

**Fig 2:**
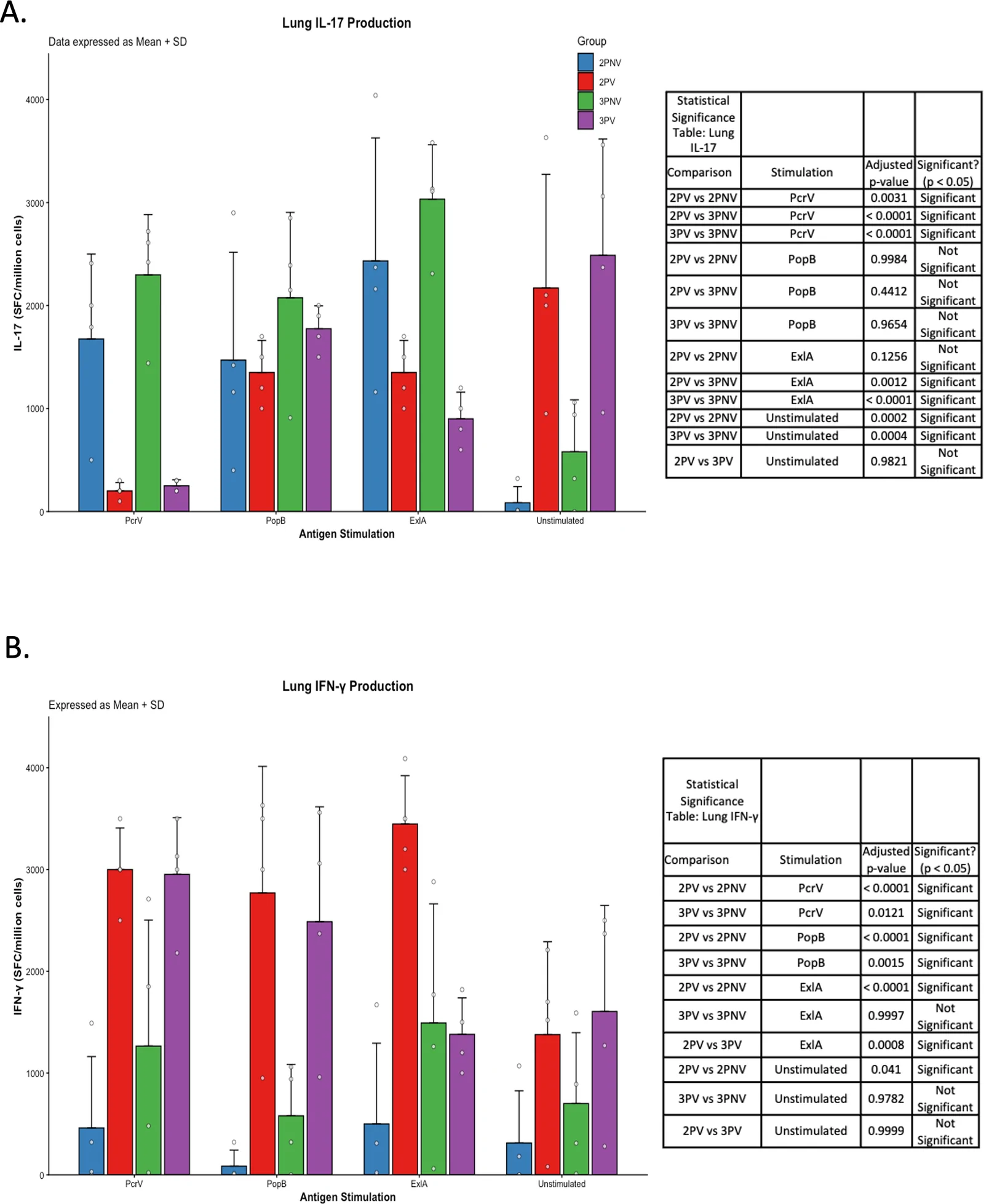
Pre-exposure and vaccination frequency modulate pulmonary Th17 and Th1 memory T-cell responses. Lung cells were harvested from mice following the final immunization and prior to challenge. Single-cell suspensions were stimulated *in vitro* with recombinant antigens (PcrV, PopB, or ExlA) or left unstimulated. The frequency of cytokine-producing T cells was quantified by ELISpot and expressed as SFC per 10^6^ cells. (**A**) IL-17-producing cells and (**B**) IFN-γ-producing cells. Experimental groups consisted of mice pre-exposed two times and vaccinated (2PV) or non-vaccinated (2PNV), and mice pre-exposed three times and vaccinated (3PV) or non-vaccinated (3PNV). Bars represent the mean + SD of *n* = 4 biological replicates per group, with individual data points shown. Statistical comparisons were performed using two-way ANOVA followed by Tukey’s post hoc test. Significant differences between groups (*P* < 0.05) are detailed in the accompanying statistical summary tables.

To further establish the extent to which global lung cytokine responses were attributable to vaccination and whether vaccination augmented pre-existing T-cell immunity within the lung mucosa, we quantified antigen-specific IFN-γ and IL-17-producing cells using an ELISpot assay. Our ELISpot analyses revealed that vaccination induced a significant increase in the frequency of both IFN-γ-producing (Th1) and IL-17-producing (Th17) cells (Fig. 2), which was consistently observed in both vaccinated groups. This expansion was supported by large effect sizes; for instance, vaccination in the 2× pre-exposed group (2PV) resulted in a huge effect size increase in pulmonary IFN-*γ* spot-forming cells (*d* = 4.44) compared to non-vaccinated controls. Mice pre-exposed to Pa two (2PV) or three times (3PV) were then compared to their respective pre-exposed, but non-vaccinated control counterparts (2PNV and 3PNV) (Fig. 2A and B). The expanded pool of antigen-specific T cells is expected to enhance the capacity of the lungs to mount a faster and more robust cytokine response upon re-exposure to the pathogen in an antigen-dependent manner. Collectively, these findings indicate that vaccination elicits a robust expansion of pulmonary Th1 and Th17 cell populations, thereby establishing an enhanced state of antigen-specific cellular immunity within the lung characterized by high biological magnitude. This ability to rapidly mobilize targeted protective responses is not readily apparent after pre-exposure if subsequent vaccination does not occur.

### Vaccination induces a globally transformed and coordinated immune signature

**Fig 3:**
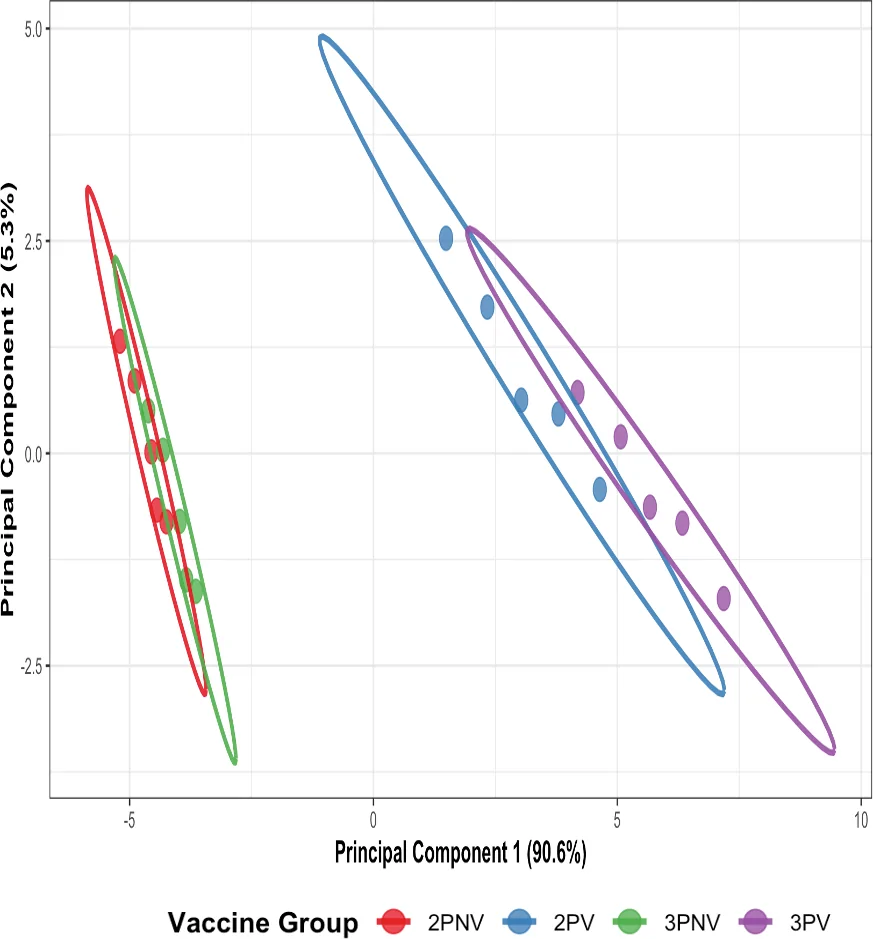
Vaccination induces a globally transformed pre-challenge immune signature. PCA was performed on the complete set of pre-challenge immunological parameters, including antibody titers and antigen-specific T-cell frequencies and cytokine responses. The score plot displays individual mice from each experimental group, color-coded, and enclosed by a 95% confidence ellipse. The plot shows clear segregation of vaccinated (PV) and non-vaccinated (PNV) groups primarily along PC1, which accounts for 90.6% of the total variance, indicating that vaccination status is the dominant factor shaping the host immune landscape.

To assess the global immunological landscape shaped by vaccination, we employed an unbiased, multivariate Principal Component Analysis (PCA), integrating all measured pre-challenge immunological features (Fig. 3). This analysis resulted in a clear segregation of the vaccinated groups from the non-vaccinated control groups. The observed separation was primarily driven by Principal Component 1 (PC1), which accounted for 90.6% of the total variance, indicating that this component captured the dominant sources of variability within the data set. This dominance of PC1 indicates that the vaccination status was the defining factor of the host immune state, eclipsing the more subtle variance attributable to the number of prior exposures. All vaccinated mice (2PV and 3PV) formed distinct clusters on the positive side of PC1, which were clearly separated from the non-vaccinated control cohorts (2PNV and 3PNV). These findings indicate that vaccination elicited a reproducible alteration in the global host immune profile, independent of the number of prior antigen exposures.

**Fig 4:**
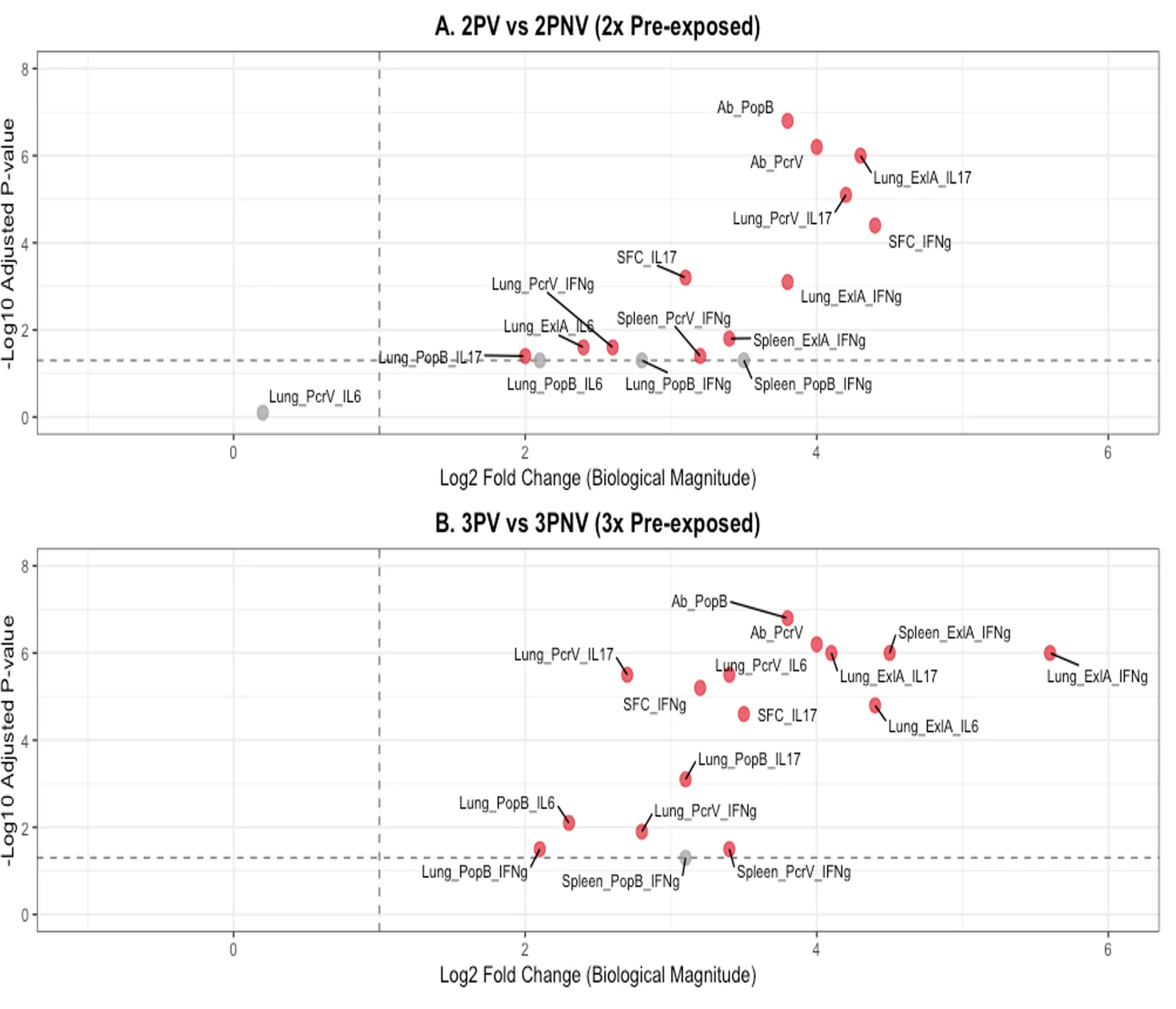
Vaccination coordinately upregulates a broad array of humoral and cellular immune responses. Volcano plots illustrate the statistical significance versus the biological magnitude of change for all measured immune parameters. (**A**) Comparison between the vaccinated (2PV) and non-vaccinated control (2PNV) groups. (**B**) Comparison between the vaccinated (3PV) and non-vaccinated control (3PNV) groups. Figures were prepared by calculating the log2-transformed ratio of the means for each parameter (*x*-axis), providing a standardized measure of the biological effect size (fold change). Statistical significance was determined using a two-way ANOVA followed by Tukey’s post hoc test; the resulting adjusted *P*-values were then −log10-transformed to define the *y*-axis. Points in the upper-right quadrant represent parameters significantly upregulated following vaccination. Dashed lines indicate predetermined thresholds for statistical significance (*P* < 0.05) and biological effect magnitude (log2 fold change = 1, representing a minimum twofold increase). Parameters exceeding both thresholds are highlighted in red, demonstrating a robust and comprehensive upregulation of the immune signature regardless of pre-exposure frequency.

To deconstruct the specific contributions driving the global shift shown in Fig. 3, we generated volcano plots comparing the vaccinated groups against their respective non-vaccinated controls (Fig. 4). This analysis confirmed that vaccination elicited a broad and robust immunological response, characterized by the significant upregulation of numerous immune mediators. Their localization within the upper-right quadrant of the plots reflected both high statistical significance (adjusted *P* < 0.05) and substantial biological magnitude (effect size). In mice exposed to *Pa* twice (Fig. 4A) and three times (Fig. 4B), vaccination elicited significant upregulation across nearly all measured immunological parameters, with many exhibiting a greater than a fourfold increase (log2 fold change >2). This included increases in antigen-specific antibody titers against both PcrV and PopB, and a potentiation of antigen-specific cytokine secretion (IFN-*γ*, IL-17A, and IL-6) from both the spleen and the lung, reinforcing the Th1/Th17 polarization observed earlier.

The macroscopic immune shift visualized by the PCA was mechanistically driven by the concerted and synergistic upregulation of individual immune components revealed in the volcano plots. Vaccination not only boosts a single parameter but also orchestrates a systemic and local elevation of both humoral and cellular immunity, creating a new and potentially heightened state of immunological readiness poised for rapid and effective pathogen clearance.

### Vaccine-primed immunity results in strain-specific and exposure-dependent impacts on bacterial clearance

**Fig 5:**
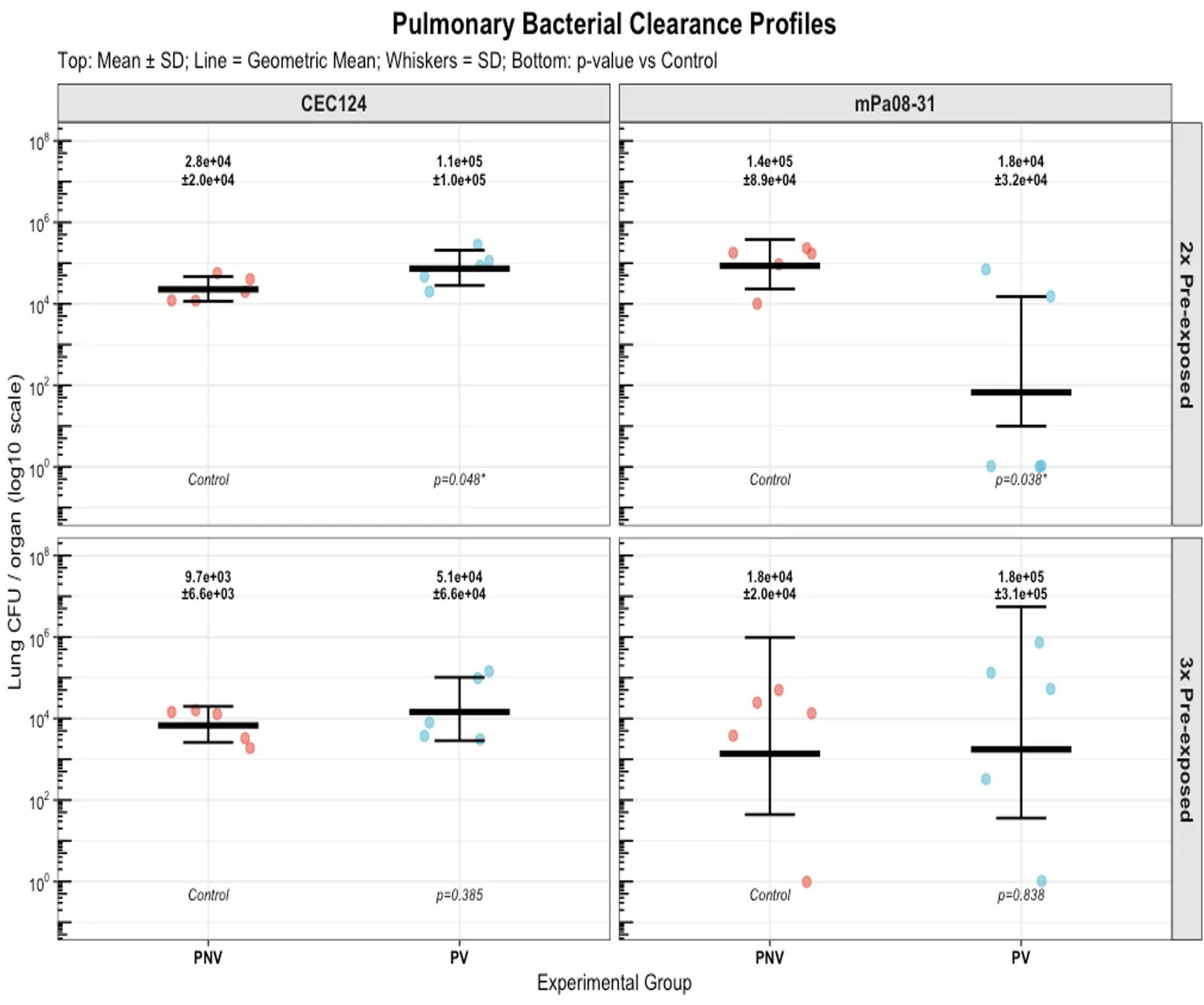
Vaccination promotes strain-specific pulmonary bacterial clearance in pre-exposed hosts. Mice were pre-exposed to *Pa* mPa08-31 either two times (2×) or three times (3×) before receiving the multivalent vaccine (PV) or remaining non-vaccinated (PNV). Lungs were subsequently challenged with the T3SS-negative/ExlA-positive strain CEC124 or the T3SS-positive strain mPa08-31. Bacterial burden (CFU/lung) was quantified at 24 h (CEC124) or 48 h (mPa08-31) post-infection. Data are presented on a log10 scale, with thick horizontal lines representing the geometric mean and whiskers denoting the SD. Individual biological replicates (*n* = 5) are shown as circles. Upper text denotes the raw Mean ± SD for each group. Statistical significance was determined using a two-way ANOVA followed by Tukey’s post hoc test on log10-transformed values. Adjusted *P*-values provided at the bottom of the panels represent the comparison of each vaccinated (PV) group against its respective non-vaccinated (PNV) control group. *P < 0.05.

The ultimate measure of vaccine efficacy is its ability to confer protection against pathogens. Therefore, we evaluated whether vaccination enhanced the capacity of mice to control pulmonary infections with two Pa strains representing distinct virulence paradigms: mPa08-31 (mucoid, T3SS-positive) and CEC124 (non-mucoid, T3SS-negative/ExlA-positive). To account for the exponential nature of bacterial replication and to stabilize variance, CFU counts were log10-transformed prior to analysis using unpaired *t*-tests. The results demonstrate that the effect of vaccination on bacterial clearance is highly dependent on both the virulence profile of the challenge strain and the host exposure history (Fig. 5).

A clear protective effect of vaccination was observed in the twice-exposed cohort (2PV) when challenged with the T3SS-positive mPa08-31 strain. Vaccinated mice exhibited significantly lower pulmonary bacterial burden than non-vaccinated controls (2PNV; *P* = 0.038), with a large biological effect size (Cohen’s *d* = 1.74) (Fig. 5B). In contrast, in the thrice-exposed groups, both vaccinated and non-vaccinated animals (3PV and 3PNV) displayed similarly efficient bacterial clearance (*P* = 0.838, *d* = 0.13) (Fig. 5D). These findings suggest that repeated natural exposure to the T3SS-positive strain establishes a protective “threshold” that diminishes the additional measurable benefit of vaccination against that specific strain.

In contrast, vaccination did not confer any protection against the T3SS-negative CEC124 strain. In the twice-exposed cohort, vaccinated animals (2PV) displayed significantly higher pulmonary bacterial burdens than non-vaccinated controls (2PNV; *P* = 0.048), with a large effect size (*d* = 1.41) and considerable inter-individual variability (SD ≈ 1.0 × 10^5^ CFU) (Fig. 5A). A similar lack of protective effect was observed in the thrice-exposed cohort (*P* = 0.385, *d* = 0.58). The increased pathogen load in vaccinated mice relative to controls following CEC124 challenge suggests that the vaccine-induced immune response may not effectively target the virulence determinants associated with this strain and may potentially alter the protective mechanisms generated through prior natural exposure.

**Fig 6:**
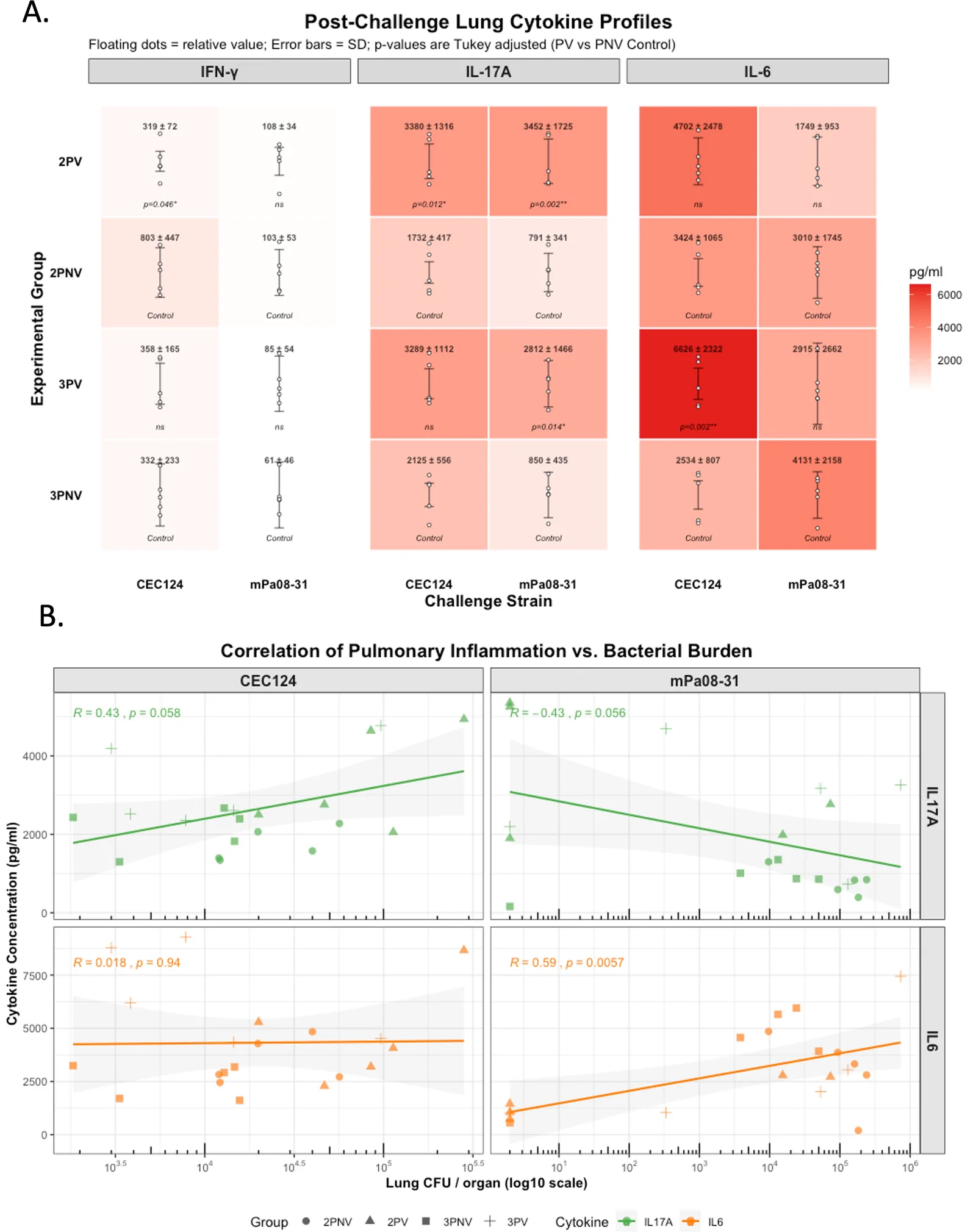
Evaluation of the post-challenge pulmonary inflammatory landscape and its relationship to pathogen control. Lung tissue was harvested from mice at 24 h (CEC124) or 48 h (mPa08-31) post-infection for cytokine quantification via multiplex immunoassay. (**A**) Integrated profile of post-challenge cytokine production. Data are visualized as a heatmap where background color intensity represents the group mean concentration (pg/mL) for IFN-*γ*, IL-17A, and IL-6. Each tile provides a detailed statistical summary: the upper text indicates the mean ± SD; central white circles represent individual biological replicates (*n* = 5) positioned vertically according to relative concentration with whiskers denoting the SD; and the lower text displays the Tukey-adjusted *P*-value. Statistical significance was determined by two-way ANOVA followed by Tukey’s post hoc test, comparing vaccinated (PV) groups to their respective non-vaccinated (PNV) control groups. (**B**) Correlation of peak inflammatory magnitude and pulmonary bacterial burden. Scatter plots illustrate the relationship between local inflammatory cytokine levels (IL-17A, green; IL-6, orange) and lung bacterial burden (CFU/organ). Pathogen burden is plotted on a log10 scale to reflect the multiplicative nature of bacterial growth. Individual points (*n* = 20 per panel) represent single biological replicates across all experimental groups. Solid lines indicate linear regression with shaded 95% confidence intervals. Pearson correlation coefficients (*R*) and *P*-values are indicated, revealing distinct strain-specific inflammatory trajectories post-challenge. *P < 0.05, **P < 0.01.

To further investigate the local inflammatory environment, we measured pulmonary cytokine responses following the challenge (Fig. 6). Elevated pulmonary concentrations of IL-17A and IL-6 were detected across the experimental groups, indicating robust inflammatory activation following challenge. Notably, in the CEC124 2× cohort, the higher bacterial burden in 2PV mice was associated with a significant reduction in IFN-*γ* production compared to that in non-vaccinated controls (*P* = 0.046), suggesting that vaccination may alter the Th1-associated response during infection with the ExlA-positive strain. Furthermore, the highest inflammatory magnitude of inflammation was observed in the 3PV cohort following CEC124 challenge, characterized by a massive pro-inflammatory IL-6 burst (6,626 ± 2,322 pg/mL; *P* = 0.002) (Fig. 6A).

Correlation analysis revealed that the relationship between cytokine magnitude and bacterial burden was strain-specific (Fig. 6B). Following the mPa08-31 challenge, a significant positive correlation was observed between IL-6 and bacterial load (*R* = 0.59, *P* = 0.0057), indicating that higher pathogen burdens directly drove the pro-inflammatory output for this strain. In contrast, no significant linear relationship was observed for the CEC124 challenge (*R* = 0.018, *P* = 0.94). These findings suggest that protection is not merely a function of peak inflammatory magnitude during an established infection but rather hinges on the qualitative features and speed of the initial immune recall established by the vaccine prior to challenge.

### Post-challenge inflammatory trajectories are associated with distinct infecting strain virulence profiles

**Fig 7:**
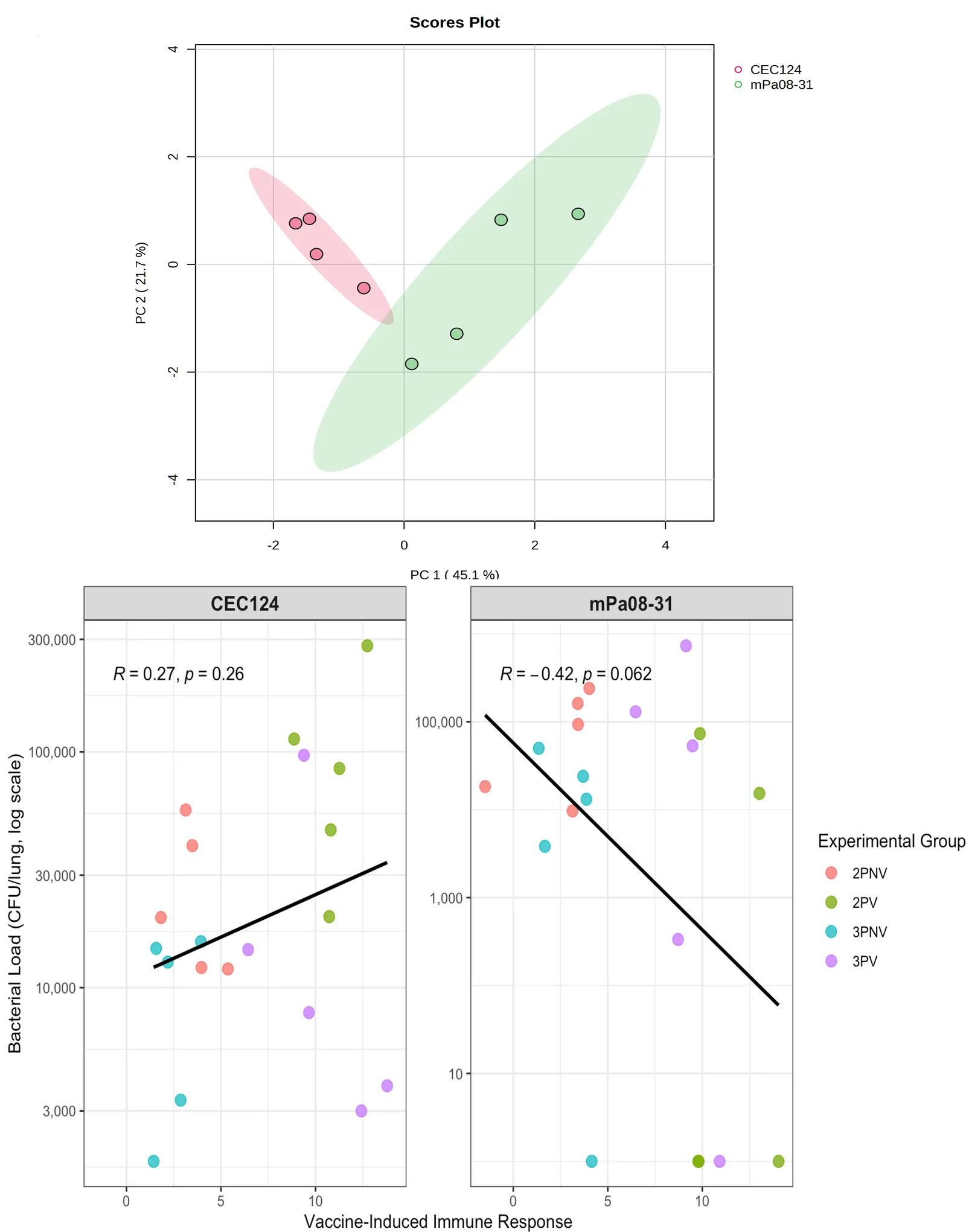
Characterization of post-challenge inflammatory environments and correlates of protection across distinct challenge strains. (Top) PCA scores plot of post-challenge lung cytokine profiles. Individual biological replicates are plotted, revealing clear clustering based on the infecting strain (CEC124 in red; mPa08-31 in green), irrespective of vaccination status. PC1 accounts for 45.1% of the total variance, indicating that the infecting strain is the dominant factor shaping the acute inflammatory environment. (Bottom) Correlation analysis between the pre-challenge “Vaccine-Induced Immune Response” (an integrated proxy of humoral and cellular immunity) and the post-challenge pulmonary bacterial burden (CFU/lung). Pathogen burden is presented on a log10 scale. Individual data points represent single mice color-coded by experimental group. Solid lines indicate linear regression. The Pearson correlation coefficient (*R*) and exact *P*-value are provided for each challenge. A strong trend toward a negative correlation (*R* = −0.42, *P* = 0.062) was observed for mPa08-31, suggesting that vaccine-augmented immunity is a putative correlate of protection against T3SS-positive strains. In contrast, the lack of correlation for CEC124 (*R* = 0.27, *P* = 0.26) highlights the impact of immunological interference when facing ExlA-positive, T3SS-negative pathogens.

Our data suggest that the post-challenge inflammatory environment is heavily dictated by the specific virulence factors of the infecting strain. This observation implies that the functional outcome of vaccine-augmented immunity is highly context-dependent, where the effectiveness of a Th1/Th17 recall is constrained by the pathogen’s specific mode of infection (e.g., T3SS-positive vs ExlA-positive). To dissect the interplay between host immunity and pathogen-specific virulence in the post-infection environment, we performed a multivariate PCA on the lung cytokine profiles measured after the challenge (Fig. 7, top panel). This analysis revealed that the intrinsic properties of the infecting strain were the single most dominant factors shaping the host’s acute inflammatory response. Mice challenged with CEC124 formed a distinct and non-overlapping cluster from those challenged with mPa08-31, with a clear separation along principal component 1 (accounting for 45.1% of the total variance). This finding suggests that the two strains elicit fundamentally different local immune environments, or “inflammatory trajectories,” a divergence that overrides the host’s pre-existing vaccination status once the infection is established.

Having established these strain-specific inflammatory signatures, we next determined whether the pre-challenge immune potentiation conferred by the vaccine was correlated with a positive protective outcome. To achieve this, we plotted the pre-challenge “Vaccine-Induced Immune Response” proxy, a value integrating the coordinated humoral and cellular immunity established by vaccination (from Fig. 4), against the post-challenge pulmonary bacterial burden, which was log10-transformed for analysis (Fig. 7, bottom panels). For the mPa08-31 challenge, a strong trend toward a negative correlation was observed (*R* = −0.42, *P* = 0.062). This suggests that a more robust vaccine-induced immune state is predictive of enhanced bacterial clearance and is therefore a putative correlate of protection against T3SS-positive strains. In contrast, no such relationship was observed for the CEC124 challenge (*R* = 0.27, *P* = 0.26).

This lack of correlation, paired with the high individual variability observed in the post-challenge cytokine profiles (mean ± SD in Fig. 6A), suggests that while the vaccine successfully established a potent Th1/Th17 response, the virulence mechanisms of the CEC124 strain rendered it less susceptible to this specific mode of immune attack. Collectively, these data indicate that the success of vaccine-conferred immunity is contingent upon a “lock-and-key” matchup between the host’s prepared defenses and the pathogen’s specific vulnerabilities. While the vaccine establishes a potent immune memory, the functional outcome of that memory is ultimately dictated by the nature of the challenge strain.

## DISCUSSION

In an era where the spectrum of antimicrobial resistance looms large, the need to develop novel preventative strategies against high-priority pathogens, including Pa, cannot be overstated. The limitations of serotype-dependent vaccines have necessitated a paradigm shift toward approaches that elicit broad immunity against highly conserved virulence factors (21). Our study contributes to this effort by providing a comprehensive pre-clinical validation of a multivalent subunit vaccine, not in immunologically naive animals, but in a more clinically relevant model simulating prior bacterial exposure. The central finding of this study is that our rationally designed multivalent vaccine significantly engages and augments a host’s suboptimal, pre-existing immunity to provide a statistically significant reduction in pulmonary bacterial burden. By utilizing log10 transformation to stabilize the variance and accurately reflect the exponential nature of bacterial growth, we demonstrated that vaccination significantly enhanced the clearance of the T3SS-positive strain in the twice-exposed cohort.

A cornerstone of the success of our vaccine’s lies in its ability to orchestrate a highly coordinated Th1/Th17-polarized immune response. Our data demonstrated that vaccination dramatically increased the frequency and functional capacity of both IFN-γ (Th1) and IL-17A (Th17) producing T cells, both locally in the lung and systemically in the spleen. This specific dual response is widely considered ideal for controlling Pa (22). Crucially, these changes were not merely significant by *P*-value but were characterized by exceptionally large biological effect sizes (Cohen’s *d* values reaching 21.35), indicating a profound transformation of the immune landscape rather than a negligible shift. The specific cytokine signature elicited in vaccinated mice may be of profound immunological significance for controlling Pa. The pronounced secretion of IFN-γ is the hallmark of a robust Th1 response, which is essential for activating macrophages to enhance their bactericidal capacity (23). Concomitantly, the high levels of IL-17A signify strong Th17 polarization (24), a pathway paramount for recruiting and activating neutrophils to mucosal surfaces—the primary effector cell for clearing extracellular bacteria (25, 26). Given the intranasal route of immunization, this robust Th1/Th17 response is likely mediated by the induction of lung tissue-resident memory (TRM) T cells. Literature suggests intranasal subunit vaccines effectively populate the respiratory mucosa with Th17-polarized TRM cells, which act as immediate “first-responders” to provide site-specific protection (27). Our recent characterization of the L-PaF/ME/BECC platform confirmed that this specific formulation induces a functional population of CD4+ lung TRMs (CD44+CD62L−CD69+CD103+) (28). By utilizing FTY720-mediated blockade of circulating lymphocytes, we established that these vaccine-induced TRM cells are sufficient to drive early pulmonary clearance of *Pa* independent of systemic recruitment. The massive, antigen-specific secretion of IL-17A and IFN-*γ* in the lungs of our currently vaccinated mice (Fig. 1B) strongly indicates the mobilization of this functional TRM signature. This resident memory provides the lung with a state of heightened readiness that natural pre-exposure alone may be unable to achieve. The co-expression of the pleiotropic cytokine IL-6 further supports this potent inflammatory state (29), particularly in the thrice-exposed vaccinated group where the vaccine-induced IL-6 boost exhibited a huge effect size (*d* = 11.31), suggesting that natural exposure history dictates the magnitude of the subsequent vaccine-augmented recall (30, 31). Complementing this cellular recall, vaccination significantly potentiates the humoral arm. While pre-exposure establishes a baseline dominated by anti-LPS titers (Fig. S1A), vaccination provides the high-titer IgG against PcrV and PopB (Fig. 4) required for a balanced defense. Furthermore, although mucosal sIgA was not measured here, the massive local production of IL-17A (Fig. 1B) provides a strong mechanistic indicator of enhanced humoral mucosal transport. IL-17A is a critical driver of the polymeric immunoglobulin receptor (pIgR) required to move IgA across the epithelial barrier (32). Collectively, our data demonstrate that vaccination transforms the host’s skewed, suboptimal baseline into a superiorly balanced immune environment—combining high-titer circulating IgG, cellular TRM recall, and the Th17-signals necessary for mucosal barrier function.

The absence of a coordinated, multifaceted cytokine response in the non-vaccinated control groups, despite their history of prior exposure, underscores the transformative impact of the vaccination. While pre-exposure establishes a baseline response, this immunity remains sub-optimal and is not adequately poised for a rapid, high-magnitude effector response, as evidenced by the negligible effect sizes (*d* < 0.2) observed in our unstimulated baseline cultures. Vaccination not only expands the T-cell pool but also fundamentally enhances its functional quality, creating a dual Th1/Th17 immune arsenal. Crucially, the establishment of this potent effector capacity, both systemically, as observed in the spleen (ensuring long-term surveillance and preventing dissemination), and locally, as seen in the lung (providing immediate pathogen containment), indicates comprehensive immunological preparedness that is directly attributable to vaccine intervention compared to the non-vaccinated baseline.

Our experimental design, simulating prior, escalating encounters with Pa, is clinically salient in that vulnerable patient populations are rarely immunologically naïve. The profound and global shift in the host immune signature is clearly demonstrated by the PCA, where vaccination status accounted for over 90% of the variance, showing that the vaccine-induced response overrides the baseline state. The lack of protection against the ExlA-positive strain CEC124 in pre-exposed mice—despite the vaccine’s known efficacy in naïve mice (13)—suggests a form of “Original Antigenic Sin.” The robust recall response to PcrV and PopB appears to dominate the immunological landscape, potentially sequestering resources or suppressing the primary response to the ExlA antigen. This finding is a cautionary note for the clinical deployment of multivalent vaccines in populations with existing *Pseudomonas* colonization. This immunological transformation translates into strain-specific effects on *in vivo* bacterial clearance. Vaccinated mice demonstrated significantly enhanced clearance of the T3SS-positive strain (mPa08-31) in the twice-exposed cohort (*P* = 0.038, *d* = 1.74), providing a key validation of the efficacy of the vaccine. However, in the thrice-exposed cohort, vaccination did not further enhance bacterial clearance beyond the levels observed in the non-vaccinated controls. Our results highlight a critical “ceiling effect” in vaccine-mediated protection within this group. In the 3× pre-exposure model, natural immunity, likely driven by the robust and progressive rise in anti-LPS titers (Fig. S1A), reached a threshold sufficient to control mPa08-31 infection even without vaccination. However, vaccination fundamentally and qualitatively transformed the immune signature from a waning, suboptimal state into a robust Th1/Th17 profile. This suggests that while the vaccine provides a clear survival advantage to those with lower baseline immunity (the 2× cohort), its role in highly exposed populations may be to redirect the immune response toward a more durable and multifaceted cellular memory rather than simply increasing the magnitude of clearance. This “ceiling effect” also helps explain why the bacterial burden in the 2PV cohort was numerically lower than in the 3PV cohort; in the twice-exposed host, the vaccine-induced response provides the primary engine for clearance, whereas in the thrice-exposed host, the clearance is dominated by pre-existing anti-LPS immunity that may mask the incremental benefit of the cellular recall. This observation has significant translational implications, as it suggests that the primary utility of our subunit vaccine may be in individuals with “sub-threshold” prior immunity (e.g., those with only occasional or low-dose exposures). In these hosts, the vaccine provides the critical “boost” necessary to cross the threshold into effective protection, whereas in highly colonized or frequently exposed hosts, the vaccine may primarily serve to “re-focus” the quality of the response rather than simply increasing its magnitude.

Despite comparable bacterial clearance in some cohorts, the disparate inflammatory trajectories visualized by PCA underscored the distinct host-pathogen dynamics elicited by each challenge strain. The strong trend toward a negative correlation between the pre-challenge immune response and bacterial load for the mPa08-31 challenge (*R* = −0.42, *P* = 0.062) suggests that the vaccine-induced Th1/Th17 mechanisms are an effective countermeasure to the primary virulence strategy of this strain. In contrast, the lack of correlation for CEC124 suggests that the efficacy of the vaccine against this strain may be constrained by pathogen-specific immune evasion.

Although our murine model provides a compelling proof-of-concept, we recognize the limitations of a small cohort size (*n* = 4 or 5). However, the consistent observation of “Large” to “Huge” effect sizes across disparate immunological and microbiological parameters confirmed that our findings represent biologically robust phenomena. Furthermore, we clarified that the immunization dose utilized (1 μg of protein antigens) was well-tolerated and standard for intranasal subunit vaccination in mice, ensuring that the observed responses reflected a specific immunogenic profile rather than non-specific reactogenicity.

In conclusion, this study demonstrated that a multivalent, nanoemulsion adjuvanted subunit vaccine can successfully engage and re-potentiate pre-existing, suboptimal immunity. It does so by orchestrating a potent, coordinated Th1/Th17 response that provides broad protection against diverse and clinically relevant Pa strains. By proving its efficacy in multiple pre-clinical models, this vaccine strategy represents a significant and promising advance in the critical global effort to combat *P. aeruginosa* as a formidable opportunistic pathogen.

## Data Availability

All data generated in this study are shown in the manuscript.

## SUPPLEMENTAL MATERIAL

Supplemental figures iai.00175-26-s0001.docx Fig. S1 to S3.

ASM does not own the copyrights to Supplemental Material that may be linked to, or accessed through, an article. The authors have granted ASM a non-exclusive, world-wide license to publish the Supplemental Material files. Please contact the corresponding author directly for reuse.
