# Supplementary material for: A multivalent subunit vaccine augments pre-existing immunity to protect against T3SS-positive Pseudomonas aeruginosa but reveals immunological interference against ExlA-positive strains
Source: Infect Immun. 2026 Aug 4;94(9):e00175-26. doi: 10.1128/iai.00175-26 (PMC13552108; doi:10.1128/iai.00175-26)
Supplement: Supplementary file 1 [file iai.00175-26-s0001.docx]

**Supplemental Materials for . . .**

**A Multivalent Subunit Vaccine Augments Pre-Existing Immunity to Protect Against T3SS-positive *Pseudomonas aeruginosa* but Reveals Immunological Interference Against ExlA-positive Strains**

Debaki R Howlader^1, * #^, Sayan Das^2^, Satabdi Biswas^1^, Zackary K Dietz^1^, Robert K Ernst^2^, William D Picking^1^, Wendy L Picking^1, *^

^1^ Department of Pathobiology & Integrative Biomedical Sciences and the Bond Life Sciences Center, University of Missouri, USA, ^2^ Department of Microbial Pathogenesis, University of Maryland, USA.

^#^ Present address: ICMR-National Institute for Research in Bacterial Infections (ICMR-NIRBI), P-33 CIT Road, Scheme – XM, Beliaghata, Kolkata - 700010

^*^Corresponding authors: Wendy Picking, [wendy.picking@missouri.edu](mailto:wendy.picking@missouri.edu), Debaki Ranjan Howlader, [debaki.r.howlader@gmail.com](mailto:debaki.r.howlader@gmail.com)

**Supplemental Fig. S1.**

**Supplemental Fig. S1. Characterization of longitudinal humoral kinetics and pulmonary cytokine co-regulation.** **(A)** Kinetic profiles of antigen-specific IgG antibody titers (anti-PcrV, blue; anti-PopB, green; and anti-LPS, red) measured from serum longitudinally at days 14, 28, 42, and 56 post-initial exposure. Data are presented as line graphs for both the twice (2x; 2PNV) and thrice (3x; 3PNV) pre-exposure cohorts. Points indicate the absolute mean antibody titer (EU/mL) ± Standard Deviation (SD) of *n*=4 mice per group. Visualizing the absolute magnitude transparently demonstrates the kinetic evolution of the targeted protein antigens relative to the naturally acquired anti-LPS baseline. **(B)** Pearson correlation matrix demonstrating the coordinated relationship between local pulmonary cytokines (IFN-*γ*, IL-17A, and IL-6) across individual biological replicates (M1–M4). The color gradient reflects the direction and strength of the correlation (blue indicating strong positive correlation; red indicating negative correlation). Exact Pearson correlation coefficients (*R*) are embedded within each cell, revealing a tightly co-regulated local inflammatory network.

**Supplemental Fig. S2.**

**Supplemental Fig. S2. Unstimulated lung and spleen cells exhibit negligible baseline cytokine production.** To confirm the antigen-specificity of the cytokine responses observed in Figure 2, parallel cultures of single-cell suspensions from the **(A)** lung and **(B)** spleen were incubated for 48 hours without the addition of recombinant antigens. Cytokine concentrations (pg/ml) of IFN-*γ* (blue), IL-17A (green), and IL-6 (orange) in the culture supernatants were quantified by multiplex immunoassay. Data are presented as stacked bars representing the cumulative mean of the three cytokines for each experimental group (2PV, 2PNV, 3PV, 3PNV). Individual replicates (*n*=4) and their respective standard deviations (SD) are indicated by unique markers: IFN-*γ* (circles), IL-17A (triangles), and IL-6 (squares). Statistical comparisons performed via two-way ANOVA with Tukey’s post-hoc test (summarized in accompanying tables) revealed no significant differences between experimental groups, establishing a consistent baseline and demonstrating the absence of spontaneous cellular activation.

**Supplemental Fig. S3.**

**Supplemental Fig. S3. Comprehensive summary of pulmonary bacterial clearance across experimental cohorts.** Bacterial lung burden (CFU/organ) was quantified following challenge with the T3SS-negative **(left panel)** CEC124 and T3SS-positive **(right panel)** mPa08-31 strains. Data are presented on a log10 scale, with thick horizontal lines representing the geometric mean and whiskers denoting the Standard Deviation (SD). Individual biological replicates (*n*=5) are shown as colored circles. Upper text denotes the raw Mean ±SD for each group. Statistical significance was determined using a two-way ANOVA followed by Tukey’s post-hoc test on log10-transformed values. Adjusted p-values provided at the bottom of the panels represent the comparison of each vaccinated (PV) group against its respective non-vaccinated (PNV) control group, which serves as the biological baseline for evaluating vaccine-induced clearance. *p < 0.05.
